# Gender and leadership in conflict settings

**DOI:** 10.2471/BLT.21.287528

**Published:** 2022-01-01

**Authors:** Kristen Meagher, Iris Elliott, Preeti Patel

**Affiliations:** a R4HSSS and Conflict and Health Research Group, King’s College London, Strand, London WC2R 2LS, England.; b Irish Human Rights and Equality Commission, Dublin, Ireland.; c Conflict and Health Research Group, King’s College London, London, England.

The Women Leaders in Health and Conflict Initiative celebrated the 2021 International Women’s Day by convening an online roundtable to address gender equitable leadership in health in conflict-affected settings. Participants represented civil society organizations, United Nations agencies, frontline health workers and academics. They identified several key challenges to the advancement of women’s leadership.

The first of these challenges is addressing the male domination in the humanitarian sector.^1^ Participants spoke of the need to shift away from a boys’ club culture and from organizational structures that reinforce masculine ideologies. Participants also noted that the sector addresses gender equality and equity externally but focuses less frequently on its own systemic culture.

Women’s participation at the decision-making level declines from community to district to national and regional levels. Therefore, inclusive and supportive working environments are required to assist women’s participation, representation and leadership. Based on their experiences, participants expected that such environments would provide leadership opportunities for women as well as minority and vulnerable groups. Building and sustaining inclusive workplaces involve developing a cohort of early career women, supporting the careers of female national staff, providing flexible working options and implementing a zero-tolerance approach to discriminatory practices.

Humanitarian organizations have policies on gender equality and equity because donors require them. Yet it is not clear how, when and by whom these policies are implemented. Research conducted by Women Leaders in Health and Conflict Initiative identified evidence-based formal interventions such as policies, procedures, audits, training and evaluation.^1^ Roundtable participants commented on the benefits of these interventions, such as opening conversations and generating specific recommendations that can lead to immediate action. Policies such as quotas are important but insufficient. Gender needs to be embedded in organizational internal and external governance metrics with transparent data on systemic policies, practices and resources.

Roundtable participants highly valued mentorship because it provides many benefits to mentees. However, demand for mentors often exceeds supply. While mentoring is viewed as an enjoyable practice, participants noted it is considered invisible labour and is undervalued. Systemic investment in and recognition of mentorship is required. Such investment and recognition would be assisted through mentorship recognition within job descriptions, incentives including mentorship in promotion criteria, and reporting mentoring activity within organizational metrics.

Measures to progress women’s leadership are more likely to succeed if accountability is embedded within governance systems with clarity about who is responsible for outcomes across leadership levels. As well as internal organizational accountability, donors should scrutinize organizations and apply sanctions to those that do not fulfil commitments on advancing women’s leadership. We base this call for accountability on the view that the global Athena Swan Scheme ‒ a framework that encourages and recognizes commitment to advancing gender equality in higher education ‒ has succeeded because of performance-related resource penalties.^2^

To ensure accountability, organizations need to develop measurement metrics and reliable disaggregated data systems and practices, which measure progression (and regression) of women’s leadership throughout their career. A recent resource is the Humanitarian Advisory Group’s *Framework and tools for measuring women’s leadership and meaningful participation in COVID-19 responses.*^3^ Publicly reporting disaggregated data will make visible and accountable who is failing the mission, highlighting underperformance.

Women Leaders in Health and Conflict Initiative research found limited evidence on the role and impact of women’s leadership at the nexus of health and conflict.^4^^,^^5^ A move is required from often anecdotal discussion to the development of rigorous and authoritative evidence. Advocating for women’s rights can be complemented by evidencing the impact of women’s leadership on humanitarian action such as minimizing risk and better decision-making.^1^

Creating an effective model of women’s meaningful leadership in health and conflict is needed. Such leadership is participatory, inclusive, effective, recognized and acknowledged.^6^ Leadership must consider issues that affect women’s lives and address problem areas where progress is inadequate or inequities persist.^7^ The approach to women’s leadership must be inclusive, institutionalized and focused on intersectionality.^5^

Here we argue that opportunities exist for advancing women’s leadership in a domain that translates across sectors and supports global development and gender equality movements.^8^^–^^12^ We call for organizational reform and for donors to fund programmes on women’s leadership in conflict-affected areas.
